# Occupational Exposure to Hydrazine and Subsequent Risk of Lung Cancer: 50-Year Follow-Up

**DOI:** 10.1371/journal.pone.0138884

**Published:** 2015-09-22

**Authors:** Joan K. Morris, Nicholas J. Wald, Anna L. Springett

**Affiliations:** Centre of Environmental and Preventive Medicine, Wolfson Institute of Preventive Medicine, Queen Mary University of London, Charterhouse Square, London, United Kingdom; H. Lee Moffitt Cancer Center, UNITED STATES

## Abstract

**Purpose:**

Hydrazine is carcinogenic in animals, but there is inadequate evidence to determine if it is carcinogenic in humans. This study aimed to evaluate the association between hydrazine exposure and the risk of lung cancer.

**Methods:**

The cause specific mortality rates of a cohort of 427 men who were employed at an English factory that produced hydrazine between 1945 and 1971 were compared with national mortality rates.

**Results:**

By the end of December 2012 205 deaths had occurred. For men in the highest exposure category with greater than two years exposure and after more than ten years since first exposure the relative risks compared with national rates were: 0.85 (95% CI: 0.18–2.48) for lung cancer, 0.61 (95% CI: 0.07–2.21) for cancers of the digestive system, and 0.44 (95% CI: 0.05–1.57) for other cancers.

**Conclusions:**

After 50 years of follow up, the results provide no evidence of an increased risk of death from lung cancer or death from any other cause.

## Introduction

Hydrazine (N_2_H_4_) is a colourless flammable liquid with an ammonia-like odour. Hydrazine is mainly used as a foaming agent in preparing polymer foams, but significant applications also include its uses as a precursor to polymerisation catalysts and pharmaceuticals. Additionally, hydrazine is used in various rocket fuels and to prepare the gas precursors used in air bags. Hydrazine is used within both nuclear and conventional electrical power plant steam cycles as an oxygen scavenger to control concentrations of dissolved oxygen in an effort to reduce corrosion. Hydrazine is also used as a propellant on board space vehicles, and to both reduce the concentration of dissolved oxygen in and control pH of water used in large industrial boilers.

The International Agency for Research on Cancer evaluated the evidence of the carcinogenicity of hydrazine, and found that there is sufficient evidence in experimental animals but there is inadequate evidence in humans. Their overall evaluation is that hydrazine is possible carcinogenic to humans. Hydrazine was tested for carcinogenicity by oral administration to mice in several experiments, producing mammary, lung and liver tumours. When tested by oral administration or inhalation exposure in rats, it produced lung, liver and nasal tumours and a few colon tumours. In hamsters, it produced liver tumours and thyroid adenomas following oral or inhalation exposure [1–3].

Two previous reports on a cohort of 427 men who worked at a hydrazine plant in the East Midlands region of England found no significant association with hydrazine exposure and lung cancer [4–5]. In this paper we extend the follow-up to fifty years.

## Methods

This is a study of a cohort of 427 men who worked in a hydrazine plant in the East Midlands region of England between 1945 and 1971. The East Midlands is one of the nine official regions of England, is the eastern part of the Midlands, and encompassed Nottinghamshire, Derbyshire, Leicestershire, Rutland, Northamptonshire and most of Lincolnshire. The men were employed for at least six months during the period of hydrazine production. The factory provided information on name, date of birth, date of first employment, date they left the company and an estimate of the extent of hydrazine exposure. This exposure was estimated based on the knowledge of the factory works manager. Further details of the hydrazine production at the factory have been given in the previous reports [4–5].

Each type of employment was classified in one of three categories, according to the estimated degree of exposure. High exposure was associated with the direct manufacture of hydrazine or its derivatives, or involved the use of liquid hydrazine as a raw material (1–10 ppm). Moderate exposure was associated with an incidental presence in an area of the plant concerned with the manufacture of hydrazine or its derivatives (<0.5 or 1 ppm). Men in the low exposure category were unlikely to have been exposed to hydrazine more than slightly, and then only infrequently.

Men who were first exposed in the low or moderate categories and who were subsequently exposed in the high category contributed man-years at risk in the low or moderate categories initially and to the high category after their first exposure in that category. Similarly, all men who contributed man-years at risk >10 years after first exposure and for durations of exposure of two years or more also contributed to man-years at risk <10 years after first exposure and to less than two years’ duration of exposure.

The identifiable details of the men were used to flag their National Health Service records with the Health & Social Care Information Centre (www.hscic.gov.uk). Quarterly, any death certificates received by the Information Centre for the flagged men were then forwarded onto the study team. Dates and cause of death were then extracted. The men were followed up to the end of December 2012. The causes of death were coded using the International Classification of Diseases versions 8, 9 and 10 (ICD 8, ICD 9 and ICD 10).

It was possible to trace 406 (95%) of the 427 men. The 21 remaining men were included in the study until the last date they were known to have been living at their last known address, or in the case of four men for whom this date was missing, until the last date of their employment in the factory. These dates were all before July 1982 (the cut-off date for the first study).

Person-years at risk and death rates were calculated by five-year age groups and time since first exposure (<10 and ≥10 years). The expected number of deaths was calculated from the death rates of men in England and Wales in the same five-year age bands over the same period of time. The observed and expected number of deaths were compared and tested for significance with one sided Poisson tests.

The deaths rates in those men who had the greatest exposure (high exposure for more than two years) were also compared to the death rates for all other men with Kaplan-Meier product limit analysis. Analyses were carried out using STATA version 12 and Fortran.

This study received approval from the BMA Central & Ethical Committee, and from the UK Patient Information Advisory Group to collect information from their employment and their deaths certificates without consent to allow follow up of all cases. When this study was started in the early 1980s this was the process required. This study was approved by the Director of the Wolfson Institute of Preventive Medicine.

## Results

This study is carried out on 427 men that worked at a hydrazine plant in the East Midlands region of England. Their mean age at first exposure was 30 years (standard deviation (SD): 10.5), their mean length of total exposure was 6.8 years (SD: 6.3) and their mean length of follow up (from last exposure to death, embarkation, last trace or end of the study) was 31.7 years (SD: 16.4).

Table 1 shows the number of man-years under observation according to the category of exposure, duration of exposure, and the number of years since first exposure. Of the 16,422 man-years 18% at risk were in the high exposure category.

**Table 1:** Number of men exposed and man-years at risk by category of exposure, duration of exposure, and years since first exposure.

Category	Duration of exposure*	Years since first exposure	Number of men	Number of man-years
High‡	≥ 2 y	< 10	54	502
		≥ 10	54	1297
	< 2 y	< 10	78	352
		≥ 10	78	770
	All	< 10	78	854
		≥ 10	78	2067
Moderate and low‡	All	< 10	375	3365
		≥ 10	375	10137
All	All	< 10	507†	4219
		≥ 10	507	12203

* All men were exposed for ≥ 6 months.

† Men who at first had low or moderate exposures and who were subsequently highly exposed contributed man-years at risk in the low or moderate categories initially and to the high category after their first exposure in that category. The numbers of men in each category, therefore, add up to more than 427 in all, as some men contributed to more than one category. Similarly, all men who contributed man-years at risk ≥ 10 y after first exposure and for durations of exposure of ≥ 2 y also contributed to man-years at risk <10 y after first exposure and to < 2 y duration of exposure.

‡ High = men who may have been exposed to about 1–10 ppm hydrazine vapour in the ambient air; moderate = men unlikely to have been exposed to ≥ 1 ppm and probably < 0.5 ppm hydrazine vapour in the ambient air; low = men unlikely to have been exposed to hydrazine vapour.

Table 2 shows the number of deaths observed compared with the numbers expected. The observed mortality is close to that expected for lung cancer, cancers of the digestive system, other cancers, and ischaemic heart disease. For men in the highest exposure category the relative risks with greater than 2 years exposure and after more than 10 years since first exposure were: 0.85 (95% CI: 0.18–2.48) for lung cancer, 0.61 (95% CI: 0.07–2.21) for cancers of the digestive system, 0.44 (95% CI: 0.05–1.57) for other cancers, and 0.95 (95% CI: 0.47–1.70) for ischaemic heart disease.

**Table 2:** Number of deaths observed (O) and expected (E) by category of exposure, duration of exposure, years since first exposure, and cause.

				Deaths			
Cause of death	Category of exposure	Duration of exposure*	Years since first exposure	O	E	O/E	95% CI
Lung	High	≥ 2 y	≥ 10	3	3.53	0.85	0.18–2.48
cancer			All	3	3.82	0.79	0.16–2.30
(162–163)		All	≥ 10	6	4.55	1.32	0.48–2.87
			All	6	4.90	1.22	0.45–2.67
	Moderate or low	All	≥ 10	11	17.91	0.61	0.31–1.10
			All	11	18.86	0.58	0.29–1.04
	All	All	≥ 10	17	22.46	0.76	0.44–1.21
			All	17	23.75	0.72	0.42–1.15
Cancer of	High	≥ 2 y	≥ 10	2	3.27	0.61	0.07–2.21
digestive			All	2	3.48	0.57	0.07–2.08
system		All	≥ 10	2	4.33	0.46	0.06–1.67
(150–159)			All	2	4.58	0.44	0.05–1.58
	Moderate or low	All	≥ 10	20	17.21	1.16	0.71–1.79
			All	21	17.95	1.17	0.72–1.79
	All	All	≥ 10	22	21.53	1.02	0.64–1.55
			All	23	22.54	1.02	0.65–1.53
Other	High	≥ 2 y	≥ 10	2	4.59	0.44	0.05–1.57
cancer			All	2	4.79	0.42	0.05–1.51
(140–149,160-		All	≥ 10	2	6.13	0.33	0.04–1.18
161,164–239)			All	2	6.40	0.31	0.04–1.13
	Moderate or low	All	≥ 10	19	23.84	0.80	0.48–1.24
			All	20	24.71	0.81	0.49–1.25
	All	All	≥ 10	21	29.97	0.70	0.43–1.07
			All	22	31.12	0.71	0.44–1.07
IHD	High	≥ 2 y	≥ 10	11	11.57	0.95	0.47–1.70
(410–414)			All	12	12.35	0.97	0.50–1.70
		All	≥ 10	16	14.56	1.10	0.63–1.78
			All	17	15.50	1.10	0.64–1.76
	Moderate or low	All	≥ 10	43	53.77	0.80	0.58–1.08
			All	45	56.39	0.80	0.58–1.07
	All	All	≥ 10	59	68.33	0.86	0.66–1.11
			All	62	71.89	0.86	0.66–1.11
Other	High	≥ 2 y	≥ 10	11	20.15	0.55	0.27–0.98
Causes			All	12	21.32	0.56	0.29–0.98
(000–139,240-		All	≥ 10	11	25.18	0.44	0.22–0.78
409,415–999)			All	12	26.80	0.45	0.23–0.78
	Moderate or low	All	≥ 10	65	84.19	0.77	0.60–0.98
			All	69	89.19	0.77	0.60–0.98
	All	All	≥ 10	76	109.36	0.69	0.55–0.87
			All	81	116.50	0.70	0.55–0.86
All	High	≥ 2 y	≥ 10	29	43.11	0.67	0.45–0.97
Causes			All	31	45.76	0.68	0.46–0.96
(000–999)		All	≥ 10	37	54.74	0.68	0.48–0.93
			All	39	58.18	0.67	0.48–0.92
	Moderate or low	All	≥ 10	158	196.91	0.80	0.68–0.94
			All	166	207.62	0.80	0.68–0.93
	All	All	≥ 10	195	251.65	0.77	0.67–0.89
			All	205	265.80	0.77	0.67–0.88

* All men were exposed for ≥ 6 months.

For category and duration of exposure see footnotes to Table 1. IHD = ischaemic heart disease.

The only categories in which the number of observed deaths exceeded expected deaths (but not significantly) were lung cancer in men in the high exposure category for six months or more (6 observed vs. 4.55 expected), cancers of the digestive system in men in the moderate or low exposure category (20 observed vs. 17.21 expected) and ischaemic heart disease in men in the high exposure category for six months or more (16 observed vs. 14.56 expected). Half of the 6 deaths from lung cancer in men in the high exposure category were in men exposed in that category for less than two years. Two deaths from cancer of the digestive system occurred in men with exposure in the high exposure category compared to 3.27 expected.

Log rank test statistics from non-parametric survival analysis shows that men with the highest exposure did not seem to have significantly different mortality for lung cancer (p = 0.97), cancers of the digestive system (p = 0.43) and all cancers (p = 0.69) compared with all the other men in the study.

## Discussion

The highest relative risk for the men in the highest exposure category for six months or more was 1.32 (95% CI: 0.48–2.87) for lung cancer, which is comparable to the relative risk in the previous studies, 1.23 (95% CI: 0.25–3.61) in 1995 study [5] and 1.60 (0.19–5.78) in the 1984 study [4]. It might be expected that the highest relative risk would be for those men with the longest exposures (≥ 2years) and ≥ 10 years follow-up (to allow fully for the latency period of lung cancer), but this relative risk was only 0.85 (0.18–2.48). This provides some reassurance that despite the evidence of carcinogenicity in animals exposure at the levels observed in this study is unlikely to pose a serious risk, if indeed there is any risk at all, for humans. For cancer of the digestive system only 2 cases occurred amongst men with high exposures, the highest relative risk of 1,17 (0.72–1.79) corresponding to men with moderate or low exposures, similar to the corresponding relative risk of 1.24 (0.47–2.70) in the 1995 study.

The strengths of this study are that this is one of very few datasets available on exposure to hydrazine and there is now 50 years of follow up. This paper includes an additional 4758 (41%) man-years of follow-up and an additional 119 (138%) deaths compared to the previous report. However, the weakness is that it is a very small cohort so doesn’t have the power to detect very low relative risks or increased risks for specific cancer. The cancers examined in this study were limited to lung and digestive system due to the risks observed in animal studies.

It is possible that there could be a healthy worker effect in this data. It could also be that hydrazine workers in this company were not able to smoke since hydrazine is flammable and a dangerous fire and explosion hazard. This may explain the lack of increased risk for lung cancer and ischaemic heart disease.

Two reports on a cohort of aerospace workers showed workers with a greater hydrazine exposure had an increased risk of death from lung cancer (RR = 1.45;95% CI:0.81–2.39), but that the increased risks were not statistically significant and the upper 95% confidence interval excluded a relative risk of more than 3 [6–7] An earlier study on the same cohort [8], had found a significant effect, but they had assumed all test stand mechanics were exposed to hydrazine, in the later studies they used a more precise exposure assessment [7]. A recent study of a French cohort of uranium processing workers also showed a non-significant increased risk of mortality from lung cancer after exposure to hydrazine (RR = 1.21; 95% CI: 0.68–2.17) [9], Both of these results are not inconsistent with our results.

Despite the evidence of carcinogenicity in experimental animals, the level of exposures to Hydrazine that men have experienced in this study were not associated with an excess risk of cancer.

## Conclusion

The numbers of men exposed to hydrazine in this study were small. The results obtained are encouraging in that no obvious hazard from lung cancer or any other disease has appeared up to 50 years later. The IARC current evaluation that hydrazine is possibly carcinogenic to humans should not be changed by the results from this study. The results from this study do not indicate an excess cancer risk associated with Hydrazine exposure, but the power of the study is too limited to exclude small excess risks.
